# Infrared Thermography Smart Sensor for the Condition Monitoring of Gearbox and Bearings Faults in Induction Motors

**DOI:** 10.3390/s22166075

**Published:** 2022-08-14

**Authors:** Alvaro Ivan Alvarado-Hernandez, Israel Zamudio-Ramirez, Arturo Yosimar Jaen-Cuellar, Roque Alfredo Osornio-Rios, Vicente Donderis-Quiles, Jose Alfonso Antonino-Daviu

**Affiliations:** 1 CA Mecatrónica, Facultad de Ingeniería, Campus San Juan del Río, Universidad Autónoma de Querétaro, Av. Río Moctezuma 249, San Juan del Río, Querétaro 76807, Mexico; 2 Departamento de Ingeniería Eléctrica, Universitat Politècnica de València (UPV), Camino de Vera s/n, 46022 Valencia, Spain; 3 Instituto Tecnológico de la Energía, Universitat Politècnica de València (UPV), Camino de Vera s/n, 46022 Valencia, Spain

**Keywords:** machine condition monitoring, smart sensors, thermography, development boards, data processing algorithms

## Abstract

The monitoring of machine conditions is very important from the viewpoints of productivity, economic benefits, and maintenance. Several techniques have been proposed in which sensors are the key to providing relevant information to verify the system. Recently, the smart sensor concept is common, in which the sensors are integrated with a data processing unit executing dedicated algorithms used to generate meaningful information about the system in situ. Additionally, infrared thermography has gained relevance in monitoring processes, since the new infrared cameras have more resolution, smaller dimensions, reliability, functionality, and lower costs. These units were firstly used as secondary elements in the condition monitoring of machines, but thanks to modern techniques for data processing, the infrared sensors can be used to give a first, or even a direct, diagnosis in a nonintrusive way in industrial applications. Therefore, in this manuscript, the structure and development of an infrared-thermography-based smart sensor for diagnosing faults in the elements associated with induction motors, such as rolling bearings and the gearbox, is described. The smart sensor structure includes five main parts: an infrared primary sensor, a preprocessing module, an image processing module, classification of faults, and a user interface. The infrared primary sensor considers a low-cost micro thermal camera for acquiring the thermal images. The processing modules and the classification module implement the data processing algorithms into digital development boards, enabling smart system characteristics. Finally, the interface module allows the final users to require the smart sensor to perform processing actions and data visualization, with the additional feature that the diagnosis report can be provided by the system. The smart sensor is validated in a real experimental test bench, demonstrating its capabilities in different case studies.

## 1. Introduction

Research about condition monitoring of industrial machines is a very important field that looks for avoiding unexpected situations such as malfunctioning, failures, damage, shutdowns, and economical losses [1]. In this sense, induction motors are—and will remain so in future—one of the most important components in industry, representing between 80–90% of the equipment used for powering motion and that consume approximately the 40% of the total energy in industrial processes [2,3]. The application of these motors is diverse; for instance, they are used in pumps, fans, conveyors, manufacturing machines, among others [4], and therefore, they require peripheral components such as bearings, gearboxes and pulleys, that mostly are prone to fail, to be coupled with the process [5]. Because their importance, several works have been reported in the literature where the state, or condition, of a system is fed back through a sensor, and its measurements help to detect faults, or anomalies, in induction motors and their associated components [6]. Those methodologies are known as signal-based approaches, which use the information provided by sensors such as current [7], vibration [8], acoustic [9], or infrared [10], and through spectral techniques, they diagnose the faults in the motor and their associated elements. All these approaches have proven to be adequate in certain aspects, having advantages and disadvantages; in some cases, they can complement one another. However, there is no a single sensor capable of detecting the diversity of faults in the motors. For that reason, several approaches exist [11]. There are still some drawbacks and limitations, such as invasive sensors, signal processing offline, manual interpretation of sensors measurements and, as a consequence, the concept of smart sensors has gained attention [12]. A smart sensor is a sensor that integrates the necessary elements, such as electronic devices and implemented algorithms, to perform acquisition of data, storage, filtering, processing, bidirectional communication, and even decision making [13]. Thus, the smart sensors can be exploited to provide meaningful processed information about the fault, or yield a direct diagnosis, by using a primary sensor; unlike common sensors that output raw data that necessarily need processing and interpretation to be useful. Additionally, from the variety of sensors, those based on infrared thermography can complement this relatively new concept very well, analyzing the elements assembled in rotating machines or kinematic chains.

Throughout the years, several works have adopted infrared thermography for faults diagnosis of induction motors and their peripheral elements. For example, the first works took thermal images from a high-cost commercial infrared camera FLIR-A310, and performed dedicated segmentation processes offline on a PC on the hot regions of a motor to perform the faults identification [14]. Other works also used a commercial infrared camera FLIR-I60 to scan the hottest surfaces on the motors case, and performed additional steps on a PC, as pseudo-coloring from a grayscale image, segmenting the hottest regions, extracting features from a thermal pattern histogram, and classifying the faults [15]. Nevertheless, these approaches are limited by the infrared camera location, which must be always in the same strategic position, regulating conditions regarding the motor, such as as closed rooms without wind flow, or camera calibration through temperature sensors invading the motor case; other adjustments must also be considered, such as relative humidity, the angle of infrared emissivity, among others [14,15]. Posteriorly, in [16] a high-cost commercial thermal video camera, FLIR-SC655, was used to acquire image frames from the steady state of the motor operation, which were subsampled and windowed to avoid redundant information. Here, images features were extracted by differencing thermal histograms through standard deviation of the temperature, Gini coefficient, and moment of light. Thus, the faults classification was made using a random decision forest (RDF) classifier. The main drawbacks for this approach were the location of the camera in a dark place, to avoid additional noise, and the use of two thermocouples to measure ambient temperature for calibration, and the processing was performed offline. For its part, the authors of [17] developed the method of area selection of states (MoASoS) used to extract features from image frames obtained from a medium-cost commercial thermal video camera FLIR-E4 using its software under laboratory conditions. In this methodology, the magenta spectrum is first obtained, then feature vectors are constructed by using MoASoS and histograms, and finally, the classification of faults for an induction motor is made through Gaussian mixture models (GMM) and K-nearest neighbor (KNN). However, the efficiency of this method relies on the specified value for the threshold of binarization. On the other side, the research described in [18] presents an approach that uses pseudo-coloring process and statistical indicators such as means and standard deviations directly from the images acquired through a high-cost commercial infrared camera FLIR-E60. This approach also has the limitation of the camera position focusing the zone of the fault manifestation and offline processing. In this same line, the investigation carried out in [19] presents a methodology that combines infrared thermography with heat transfer theory. Therefore, as the first step, a high-cost commercial infrared camera FLIR-T440 and its software are used to acquire images that will be used to build a thermal model of the motor offline; later, in a second step, the model is used to compare the main differences of thermal gradients and temperatures between the healthy condition and the faulty conditions of the machine. Recently, machine learning and deep learning algorithms are being introduced in the infrared thermography analysis of faults in induction motors. For instance, a scheme based on small labeled infrared thermal images, with the enhanced convolutional neural network (ECNN) and convolutional auto-encoder (CAE), is proposed and implemented offline in [20]. In that work, the thermal images, acquired using a high-cost commercial camera FLIR-A35, allow system conditions to be characterized, then the exponential linear unit (ELU) and stochastic pooling (SP) are used to construct the ECNN. However, a CAE is pretrained; once this has been achieved, the parameters are transferred to the ECNN, and the small labeled thermal images are used to train the ECNN to classify the faults. Last, but not least, the authors of [21] propose a new methodology named binarized common areas of image differences (BCAoID), implemented by software in the PC. Therefore, as first stage, a medium-cost commercial thermal video camera FLIR-E4 records a few seconds of the analyzed system (electric impact drill), then the video is split into several images, or frames, that in turn are grouped as a training set and a test set. Posteriorly, the BCAoID extracts features from the images that will be used in the classification stage. Finally, the faults categorization is performed by KNN and a backpropagation neural network (BPNN) for comparison purposes. As seen in previous works, the infrared thermography analysis has been evolving through the years, even though the segmentation methods are still used; today, new techniques are integrated in such a way that signal-based approaches are being substituted by model-based or data-driven approaches. However, it worth noticing that infrared thermography can still be exploited, because the smart sensor concept and data-driven techniques can be analyzed in depth and can provide novel solutions to the faults diagnosis of induction motors.

It can be said, according to several works in the state of the art, that the most frequent faults that occur in induction motors can be divided into two main branches: electrical and mechanical [22]. However, this paper focuses on mechanical faults associated with two principal elements of induction motors: rolling bearings and gearboxes. Rolling bearings are of especial interest, since through the years, these elements have been studied and several techniques have been developed for diagnosing fault conditions such as damage, or wear in the inner race, outer race, inner–outer race, balls, and lubricant [23,24,25]. The importance of rolling bearings is justified, since they are very common components in the induction motor, used for the purpose of coupling shafts inside and outside the motor. They are prone to fail due to many factors, such as changing load conditions, electric erosion by parasite currents, wear, corrosion, etc., and the cost losses can be easily scaled depending on the condition severity [23]. For its part, the gearboxes are peripheral components that serve the induction motor for coupling power motion of the process; for instance, performing a balance between power torque and speed [26]. Like any other mechanical component, the gearboxes can present faults in the internal elements, such as wear, corrosion, backlash, or broken teeth of the gears [27]. For this reason, many works have been developed to diagnose the fault condition of gearboxes [28,29,30]. The importance of the rolling bearings and the gearboxes is clear, as they are essential elements that work with the induction motor to integrate an ensemble that provides motion of an industrial process. Additionally, in both elements, the study of gradual failures is in vogue, and not only the in detection of a catastrophic or advanced state in the failure. Additionally, it is well known that monitoring systems that address fault diagnosis of rotating machinery involve the processing of a big amount of data, especially in the monitoring of critical sectors of industry that use induction motors [31]. One emerging solution for handling data is through data fusion, used to accurately perform the fault diagnosis of motors. In this field, several works have been proposed. For example, in [32] an automated framework is presented for diagnosing faults in gearboxes of rotating machines. In this framework, the frequency domain fusion of data from vibration sensors is performed, the feature extraction is performed through coherent composite spectrum, the dimensionality reduction of data is carried out by means of principal component analysis (PCA), and the fault classification is achieved by using the artificial neural network (ANN). Meanwhile, the authors of [33] describe a deep-learning-based model for fault diagnosis of motors named the multi-resolution and multi-sensor fusion network (MRSFN). In that work, the multi-scale analysis for the signals of vibration and stator current is performed by generating windows of varying lengths. Finally, through the convolutional neural network (CNN) and the long short-term memory (LSTM) the proposed approach is capable of learning discriminative features related to faults. This approach was validated considering the healthy condition of the motor, a broken rotor bar, a built-in bowed rotor, and a faulted bearing in the inner race. In a last example, the work proposed in [34] reports a multi-sensory fusion model named dynamic routing-based multimodal neural network (DRMNN). That proposed work considers the data fusion of the signals coming from the vibration and stator current sensors. Then, feature extraction is carried out based on a multimodal scheme for dimensionality reduction and for capturing invariant features. The classification of faults is executed through the dynamic routing algorithm in the decision layer of a multimodal deep learning (MDL) structure. The methodology is validated for the fault diagnosis of the healthy condition of the motor, three broken rotor bars, rotor bent, and a bearing fault in the inner race. Other works present new strategies based on data fusion of the features extracted from the raw signals from sensors, with the aim of improving the fault diagnosis of rotating machinery. For instance, ref. [35] implements a denoising auto-encoder (DAE) and a contractive auto-encoder (CAE) used for learning the features from vibration signals. Later, a locality preserving projection (LPP) algorithm fuse the learned features, which are finally trained by a SoftMax for a model that makes the diagnosis of the rotor and bearings in the machine. For its part, the work described in [36] proposes a multi-segment features fusion for fault diagnosis of motors. Here, the raw signal is divided into segments using the Grassmann manifold and the angular central Gaussian distribution, and the feature extraction is performed through the wavelet transform and the ensemble empirical mode decomposition. The high number of features is reduced through morphological processing, and the data fusion is achieved by using a deep belief network. Thus, the classification is carried out with a strategy that uses pairwise coupling combined with a sparse Bayesian extreme learning machine. As last example, the research presented in [37] performs muti-source data fusion, where some extracted features are empirical and some others are hidden features. Additionally, the CNN is implemented to obtain recessive features of the complex signal waveforms. The fusion task is considered for statistical features and for recessive features that are input to a light gradient boosting machine (LigthGBM) model for fault diagnosis in the rotor and stator of the machine. As observed, several of the works developed justify the importance of fault diagnosis in motors, because the diagnosis technique adopted is still a topic of interest.

The contribution of this work is the structure and implementation of a smart sensor based on infrared thermography for fault diagnosis of rolling bearings and gearboxes associated with induction motors. Additionally, it can monitor the motor, or the elements to be evaluated (bearings or gears), to determine what segment of the thermal image delivers the best diagnosis. Therefore, the smart sensor integrates, in its core, a primary sensor unit, consisting of a low-cost thermographic infrared camera used to obtain the thermal images, with an electronic board, which in turn consists of a microcontroller unit for data processing, and the implementation of dedicated algorithms designed for detecting and diagnosing faults conditions. Therefore, five main blocks define the functionality and operability of the smart sensor: primary sensor (infrared camera), image preprocessing, image processing, features extraction, and faults diagnosis. The algorithms implemented into the processing unit are defined to condition the thermal image by resizing it, eliminating noise, segmenting, extracting the features, and classifying the faults. Later, the smart sensor is presented in detail from the viewpoint of a functional module with specific characteristics. Posteriorly, some test and validations are presented, demonstrating the functionality of the smart sensor for three operating conditions of the elements of the induction motor: analysis of rolling bearings, analysis of gearbox coupling, and combined analysis rolling bearings-gearbox. Finally, it must be highlighted that the smart sensor provides the results of the faults and their graduality on screen through a developed user interface, but also, the diagnostic can be sent to other devices, such as a PC, through an output interface.

## 2. Structure and Design of the Infrared Thermography-Based Smart Sensor

**Figure 1:**
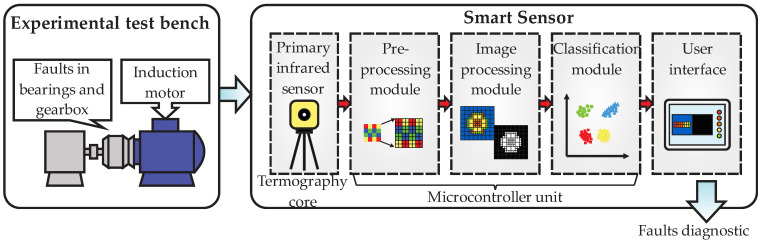
Block diagram of the general structure of the smart sensor for fault diagnosis based on infrared thermography.

This section will present the structure of the smart sensor; thus, Figure 1 presents a general block diagram of this structure showing two parts: the experimental test bench, and the smart sensor. The experimental test bench block indicates the applicability of the proposed smart sensor, which is for diagnosing faults in the associated elements of induction motors; in this sense, the motor has main rolling bearings and a gearbox that allow us to couple the motor with the process. For its part, the smart sensor block shows a core that can be divided into five main parts: (1) primary sensor, (2) preprocessing module, (3) image processing module, (4) classification module, and (5) user interface.

As it can be seen from figure, the general block diagram assumes two main parts for the study: the process to be analyzed and the faults diagnostic. The process can be any application of industry, but at least must have the induction motor, which internally has rolling bearings supporting the rotating shafts and externally should have means to be coupled to the process chain, such as a gearbox. Therefore, several aspects can affect the performance of the elements in the process, such as environmental conditions, process conditions, aging over time, wear, etc., and these situations can be reflected as a rise in the temperature of specific zones in the motor surroundings. Traditionally, faults detection has been performed considering changes in the motor operation; therefore, these variations in the temperature are also useful for detecting problems in the peripheral elements of the motor.

The smart sensor core, for its part, considers the integration of a basic infrared primary sensor for acquiring the thermal images from the process, but these data require analysis and interpretation, and this task is achieved through data processing stages. Hence, three modules are implemented into a microcontroller unit for image preprocessing, image processing, and faults classification. The preprocessing module performs the first interpretation by taking the raw data from the primary sensor and generating a digital image of temperature profiles. Next, the processing module performs a resizing of the original image to a more adequate resolution for further processing. Then, image segmentation of the hottest zones in the system is performed by assuming three main zones, or regions of interest (ROIs): motor coupling zone (gearbox zone), induction motor body, and back zone of induction motor. Once the ROIs are defined, the extraction of features is performed, and this task is achieved by computing 15 statistical indicators that will be arranged into a high-dimensionality matrix of features, with the purpose of having as much information as possible related to the faults. Posteriorly, the classification of faults present in the ROIs is performed in two steps. Firstly, as the high-dimensionality matrix of features can present repeated information, or even useless data, it is necessary to reduce this matrix through the principal component analysis (PCA) to avoid data redundancy, and to have the meaningful features related to the faults, which yields a two-dimensional graphical representation of the faults as grouped data. Secondly, the final classification of the faults is achieved by inputting the output features from the PCA in a deep neural network (DNN) structure. Last, but not least, a user interface performs the presentation of the information processed by the smart sensor through graph templates that facilitate the interpretation of the fault diagnostic. Additionally, the diagnostic report can be outputted from the smart sensor through an output port with the purpose of visualizing the diagnosis offline in the PC.

### 2.1. Experimental Test Bench

The operating conditions of the experimental test bench are described in the following lines. The test bench consists of a topology that couples an induction motor with a process through a gearbox; in this case, the process is represented by a simple load using an alternator. The induction motor is a model WEG 3F A.E. 00136AP3E48TCT that has a rating power of 0.74 kW, with nominal speed of 3355 RPM. Additionally, the induction machine is fed with a VFD model WEG CFW08 operating with a 210–230/460 Vac at 60 Hz. On the other hand, the gearbox is a model Baldor GCF4X01AA with a reduction ratio 4:1 that drives the motor shaft. As mentioned before, the process is represented by a load, through an alternator, entailing a total motor load of approximately 10%. 

#### Faults Conditions

**Figure 2:**
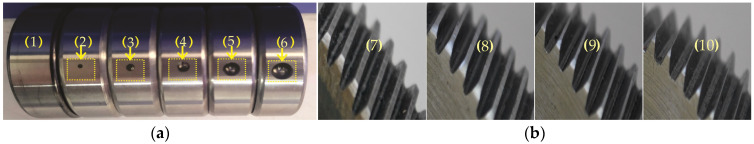
Fault conditions and their graduality, in (**a**) outer-race faults of the rolling bearings, and in (**b**) tooth wear of a gear into the gearbox.

Two main elements associated with the induction motor and its coupling to a process were considered in this work: rolling bearings and gearbox, and for each one, a fault condition and its graduality is analyzed. For the first case, we used six identical metallic bearings models 6203 2RS with eight balls, 40 mm in external diameter and 17 mm in internal diameter. Only one bearing was adopted for the healthy condition (nor fault or damage), and for each one of the remaining five bearings, an outer-race fault was induced, considering different severity. For this purpose, the fault severities were induced through a milling machine process by drilling a through-hole in the outer-race of the bearings using tungsten drill bits. Thus, Figure 2a depicts the outer-race faults considered: (1) healthy condition (no fault), (2) a hole of 1 mm diameter, (3) a hole of 2 mm diameter, (4) a hole of 3 mm diameter, (5) a hole of 4 mm diameter, and (6) a hole of 5 mm diameter. Meanwhile, for the case of the gearbox, four identical 72-teeth gears were selected for the experimentation, one assumed for the healthy condition and the remaining three with uniform wear in all the teeth. This way, three different levels of uniform wear were machined at the gear factory for the three gears, interchanged between trials, with the aim of distinguishing between different wear severities. Therefore, Figure 2b displays the gear wear conditions considered: (7) healthy condition (no wear), (8) 25% of backlash, (9) 50% of backlash, and (10) 75% of backlash.

### 2.2. Structure of the Smart Sensor

In the following lines, a detailed description of the structure, design, and implemented algorithms in the smart sensor are described. From Figure 1, it is noted that the processing and classification modules are implemented into a microcontroller unit; only the primary infrared sensor and the information presentation on screen are outside. In this sense, the microcontroller unit is a Raspberry Pi 4, 2 GB of RAM, Quad core 64-bit ARM-Cortex A72 processor that runs at 1.5 GHz. Additionally, this microcontroller unit has a VideoCore VI 3D for Graphics, supports a dual HDMI display output up to 4Kp60, has a camera port 2-Lane MIPI CSI, and supports Linux Software. In this work, the software used was Raspbian v.2022-04-04 under the General Public License (GPL) downloaded from the official website.

#### 2.2.1. Primary Infrared Sensor

The infrared primary sensor is the essential unit as the input port of the smart sensor that is used to capture thermal images from the sideway of the induction motor, and that will provide the necessary information about the faults present in the rolling bearings and the gearbox. It consists of a low-cost micro thermal camera model FLIR LEPTON 3.5 with a resolution of 160 × 120 pixels of 12 microns, thermal sensitivity < 50 mK, lens of 56°, fast time to image < 0.5 s, and a SPI video interface with two-wire serial control interface. Additionally, this micro sensor module provides calibrated radiometric output across the 19,200-pixel array, increases the scene dynamic range to +400 °C, and has automatic temperature compensation. The output format is user-selectable between 14-bit, 8-bit, or 24-bit RGB. For the proposed smart sensor, the 14-bit grayscale format is selected to generate a temperature profile map adequate for further processing. The smart sensor is placed observing the motor and the coupling sideway, allowing us to define the ROIs mentioned before.

It worth mentioning that, nowadays, there are some new-generation infrared cameras (such as the FLIR ONE) compatible with smartphones (such as the iPhone) for displaying data through apps, but they are limited by their closed processors, high costs, and restrictions in software and hardware handling. Therefore, in this proposed work, the focus of the smart sensor considers its use in industrial applications with hard environments and system characteristics such as low cost, as it is up to four times cheaper compared with some other commercial infrared cameras such as the FLIR ONE. This way, the proposed smart sensor keeps open architecture, since the functionality is defined by means of the algorithms implemented in the microcontrollers. 

#### 2.2.2. Preprocessing Module

**Figure 3:**
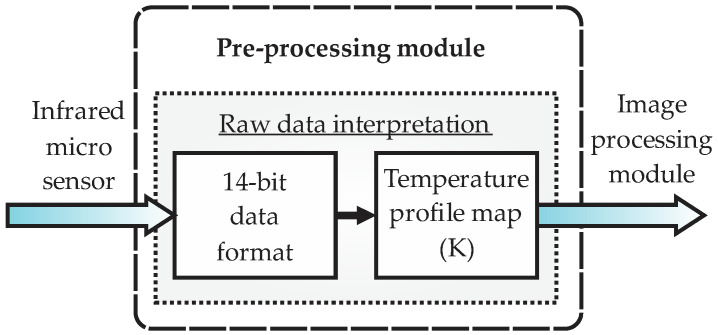
Block diagram of the preprocessing module in the smart sensor.

Once the infrared micro sensor performs the capture of the images from the process, this information is input to the preprocessing module, as observed in the detailed block diagram of Figure 3. As observed from the figure, the output from this module is a digital image; that means the raw data in a 14-bit format coming from the infrared sensor are converted to a digital image with the temperature profiles map required for processing in the next stage. 

**Figure 4:**
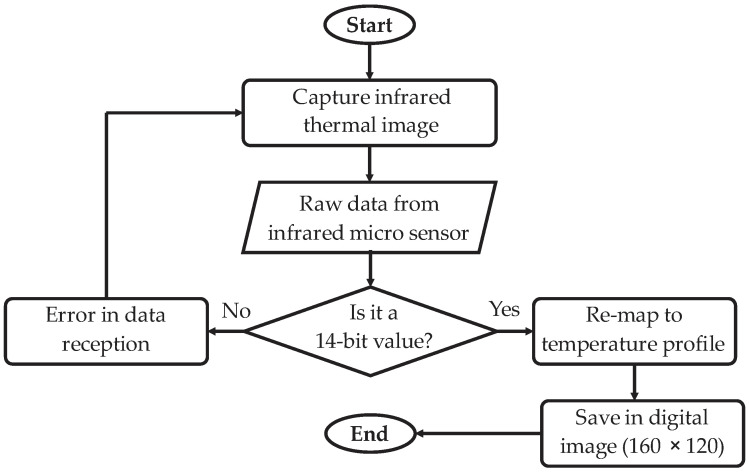
Flow diagram of the preprocessing module implementation.

The process followed to obtain the digital image from the raw data from the infrared sensor is according to the flow diagram showed in Figure 4. First, a capture of the process is performed by the infrared micro camera, then the raw data are acquired and sent to the module considering binary strings with a 14-bit format. If an acquired value does not correspond to the specified format, then an error in the data reception occurs and, consequently, the sensor must perform another capture. On the other hand, if all the received values are in the correct format, then they are mapped to a temperature profile map though the corresponding magnitude of the binary strings, yielding a digital thermal image. Finally, this image is saved with a size of 160 × 120 pixels in grayscale because the sensor has one channel. 

#### 2.2.3. Image Processing Module

**Figure 5:**
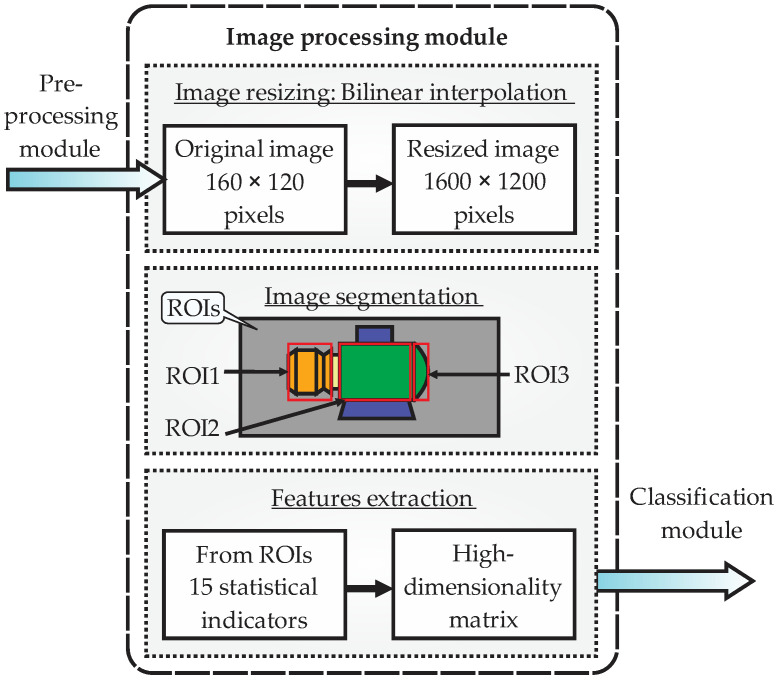
Block diagram of the image processing module in the smart sensor.

The image processing module receives the digital thermal image from the preprocessing module and generates, as output, the matrix of features of high dimensionality that will be sent to the classification module, as observed in Figure 5. This module performs three main substages: image resizing, image segmentation, and features extraction. Resizing of the original image is necessary to improve the image resolution, facilitating the segmentation process and improving the values of the features extracted. In the image segmentation, the smart sensor predefines the ROIs to be analyzed from the thermal image. Here, three main ROIs will be defined, as mentioned before: motor coupling zone (gearbox zone), induction motor body, and the back zone of the induction motor. Lastly, the features extraction is carried out by generating a matrix of features.

**Figure 6:**
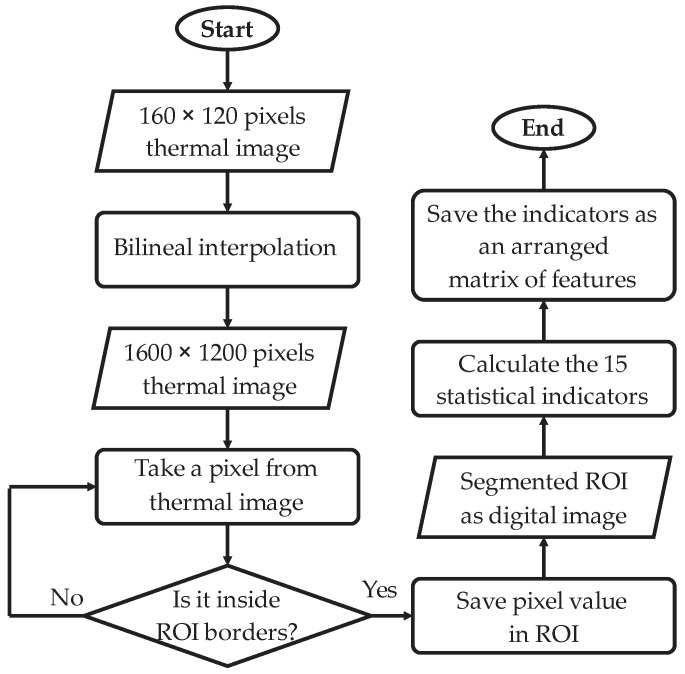
Flow diagram of the image processing module implementation.

The algorithms implemented in this module follow the flow diagram of Figure 6. As a first step, the thermal image with an original size of 160 × 120 pixels is input into the module. The second step consist of applying the bilinear interpolation to the original image, since the pixels in the thermal image are considered a two-dimensional array. This yields an image with new size of 1600 × 1200 pixels without losing resolution. In third place, the ROIs are predefined considering the three main zones previously mentioned (coupling, body, and back) through an iterative process. Thus, a pixel from the resized image is taken, then a comparison is made considering whether the pixel in question is found inside the ROI border or not. 

**Figure 7:**
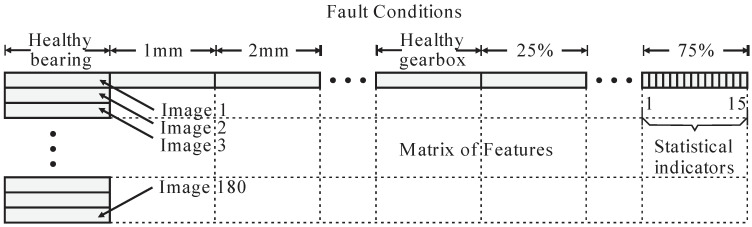
Matrix of features with high dimensionality.

If the pixel is inside the border, then it is saved in the ROI, and this process is executed three times for all the image pixels generating ROI1, ROI2, and ROI3, as new digital images. This way, ROI1 corresponds to the motor coupling zone (gearbox zone), ROI2 corresponds to the induction motor body, and ROI3 refers to the back zone of the induction motor. Once the three ROIs are defined, one set of 15 statistical indicators is computed per ROI. The statistical indicators are the mean, maximum value, root mean square, square mean root, standard deviation, variance, form factor with RMS, form factor with SMR, crest factor, latitude factor, impulse factor, skewness, kurtosis, fifth moment of inertia, and sixth moment of inertia; for more details about these indicators, see [5]. The main idea of computing these indicators is to extract as much information as possible related to the faults, so if an indicator retains valuable description related to the fault, it can be used for diagnosis. Naturally, there is a possibility that some indicators do not contain useful information, or keep duplicated information, but this drawback is overcome by the next processing stage. These indicators are arranged to form a matrix of features, which has high dimensionality with the purpose of having the features ordered (Figure 7); that is to say, this matrix will be sent to the next module.

#### 2.2.4. Classification Module

**Figure 8:**
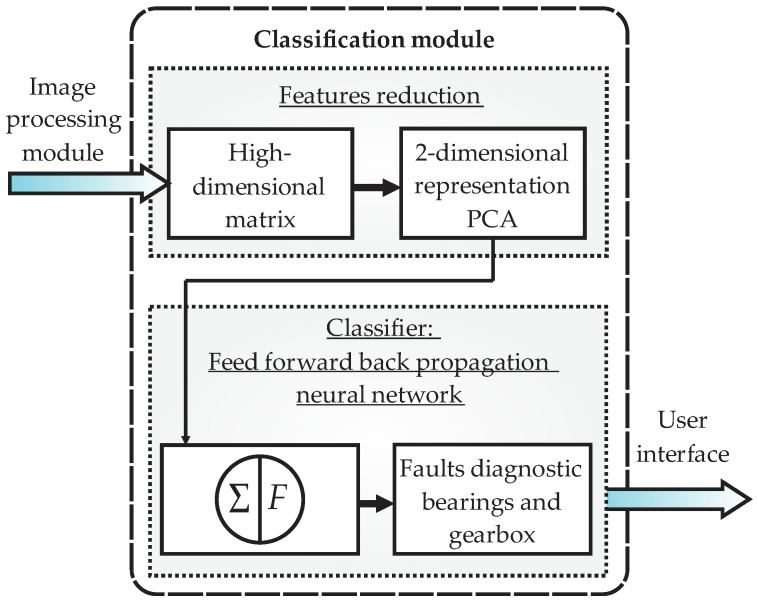
Block diagram of the classification module in the smart sensor.

The classification module, observed in Figure 8, receives as input the matrix of features of high dimensionality coming from the image processing module and generates, as output, the fault diagnostic report of the rolling bearings and the gearbox, which is sent to the user interface module. Two main substages are carried out in this module: features reduction and classification of faults. Thereupon, features reduction is performed through the principal component analysis (PCA) which is a technique used to describe data sets, better known as clusters, through new uncorrelated variables with a maximum separation between them. In general terms, the PCA looks for a projection in which the data sets will be represented in more adequate way, ordered by the value of the variance that they initially have. This reduction can be considered a preclassification stage, since it allows us to graphically visualize the conditions of the system (fault conditions) as separated grouped data (clusters). Additionally, PCA not only reduces the dimensionality of the matrix, but also avoids information redundancy, yielding only the meaningful features tightly related to the faults. As consequence, the PCA reduces the computational burden required by the classifier and reduces the error in classification because it maximizes the separation between the grouped data, avoiding the typical problems of overfitting. Then, this information is fed to the feed forward backpropagation neural network (FFBNN), whose structure has 2 neuros at the input layer, 3 and 10 neurons in the two hidden layers, respectively, and 10 neurons in the output layer (one for each fault condition). The activation function implemented is the hyperbolic tangent and the back propagation function implemented is Levenberg–Marquardt. The FFBNN is then trained and posteriorly validated for the fault conditions proposed.

**Figure 9:**
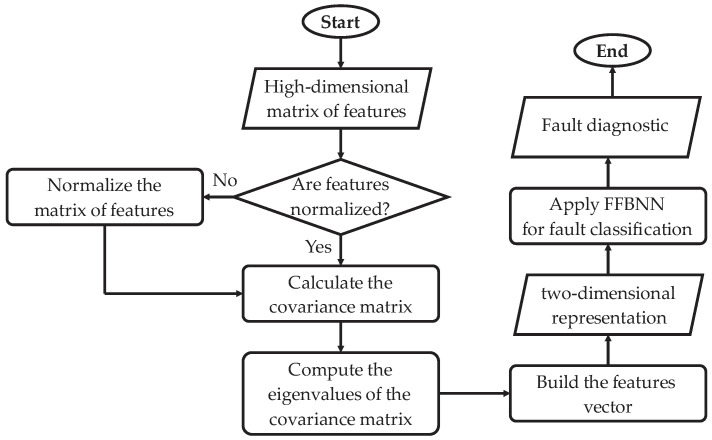
Flow diagram of the classification module implementation.

The implemented algorithms of PCA and the FFBNN are according to the flow diagram of Figure 9. Here, the high-dimensionality matrix of features is taken, then, through an iterative process, every feature in the matrix is evaluated and normalized if this has not already been achieved. Posteriorly, the PCA is applied by calculating the matrix of covariance form the normalized features; later, the eigenvalues from the matrix of covariance are computed, and finally, the features vector is built for its representation in two dimensions (two new features); for more details about this process, see [5]. Finally, the set of two features from the PCA are fed to the FFBNN, which finally classifies the faults considering the outer-race fault and gear wear, and their graduality, leading to a total of 10 conditions.

#### 2.2.5. User Interface

**Figure 10:**
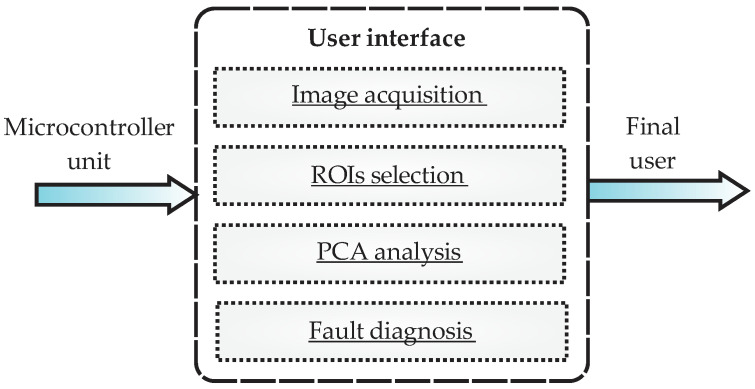
Block diagram of user interface in the smart sensor.

Last, but not least, the block diagram of Figure 10 shows the essential screens of the user interface, with which the final user can interact. In general, the information received by the user interface module comes from the microcontroller unit, and the output generated is the visualization, or presentation, of the information managed by the smart sensor though images and plots. Additionally, the user interface sends information to the microcontroller; for example, to perform the thermal image acquisition. As shown in the figure, four main tabs can be accessed by the user. The first tab corresponds to the image acquisition, the second tab corresponds to the ROIs selection, the third tab is dedicated to the PCA analysis, and the fourth tab presents the faults classifications through the FFBNN.

For each tab, some parameters can be specified by the user and others are automatically performed. For example, in the tab dedicated to the ROIs selection, the user can choose between the three predefined ROIs (coupling, body, and back) when performing the PCA and fault classification; additionally, they can perform multiple or combined analysis by using the three ROIs at the same time. This characteristic enables the smart sensor to perform fault diagnosis not only for bearings, but also for gearbox or even both conditions. For its part, the tab regarding the PCA analysis allows the user to observe the results of the matrix of features reduction, visualizing the faults grouped in clusters by using only two meaningful features. Finally, the tab regarding the fault diagnosis shows the results of the FFBNN presenting a semaphore with the 10 fault conditions, using the color green if there is no fault and the color red to indicate if there is a fault. It is worth mentioning that the report of the diagnostic can be externally obtained through an output port from the smart sensor to visualize the results in a PC offline.

## 3. Thermography-Based Smart Sensor Physical System Description

This section will describe the physical system of the smart sensor based on infrared thermography used for diagnosing faults in elements of induction motors, such as rolling bearings and their peripherals, such as the gearbox. Thus, three parts will be described: physical smart sensor, hardware integration, and user interface.

### 3.1. Smart Sensor Based on Infrared Thermography-Physical System

**Figure 11:**
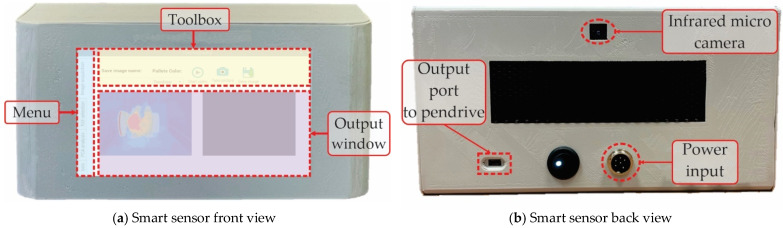
Smart sensor based on infrared thermography, physical system in (**a**) front view and in (**b**) back view.

The physical system of the smart sensor based on infrared thermography can be observed in Figure 11. Considering the basic concept of a system, the developed smart sensor interacts with the process to be analyzed (induction motor and its related elements) through its input and output ports. For example, Figure 11a shows the front view of the smart sensor with its touchscreen as an input–output port. The touchscreen displays a main menu, a toolbox menu, and an output window. These menus allow the user to require the smart sensor to perform a specific task and visualize information. On the other side, Figure 11b exhibits the back view of the smart sensor; here, other input and output ports that complement the system’s functionality are present. The infrared micro-camera is located in this place, as well as the power input and the output port used to extract the diagnostic report.

It must be highlighted that the smart sensor philosophy is that of all-in-one functionality; that means the system can be considered for commercial purposes because all the necessary elements are integrated inside the shell: sensor, processing boards, display, connectors, software, implemented algorithms, etc. Therefore, the smart sensor is a hardware–software codesign that provides an embedded system solution to the analysis of faults on industrial applications.

### 3.2. Hardware Integration of the Smart Sensor 

**Figure 12:**
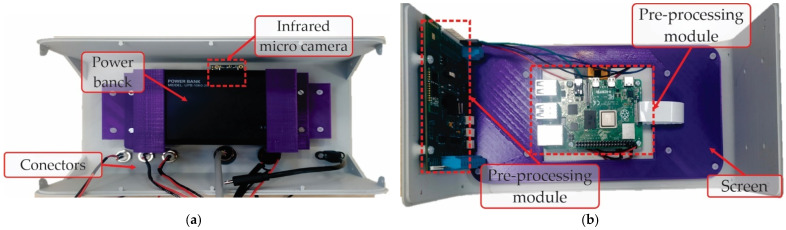
Smart sensor, physical system, inside views, in (**a**) front view and in (**b**) back view.

Regarding the hardware in the smart sensor, Figure 12 shows the main electronic components mounted and connected inside the physical system. Therefore, Figure 12a depicts the electronic boards integrated inside the smart sensors that are located in the front part. Here, the preprocessing board and the processing board are observed, as well as the mounted touchscreen (behind the processing board). It must be mentioned that the principal algorithms are coded in the processing board (Raspberry Pi 4). Meanwhile, in the preprocessing board (MSP432 from Texas Instruments), the filtering and signal conditioning are carried out. As mentioned previously, the touch screen board displays the information through the user interface graphically. In the other case, Figure 12b shows the remaining elements of the smart sensor at the back side. Between these is the power bank, which gives the system independence in the functionality, allowing us to place the smart sensor at any location around the process to be analyzed. Additionally, the infrared micro-camera and the connectors are in this place. It worth mentioning that the infrared sensor is the thermography core of the system, since the smart sensor is based in infrared thermography, and thus, the information provided by this primary sensor is vital for accurate results in the diagnosis. 

### 3.3. User Interface of the Smart Sensor

**Figure 13:**
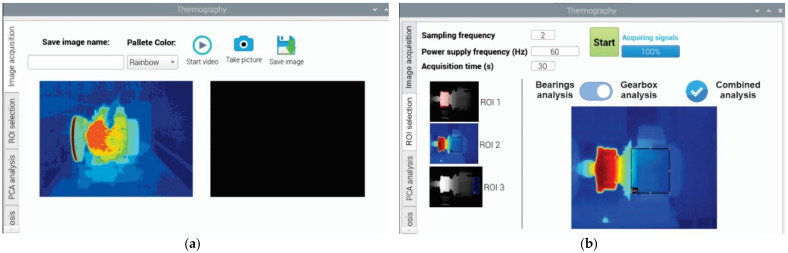
Smart sensor screen templates, in (**a**) Image acquisition tab, and in (**b**) ROI selection tab.

The user interface of the smart sensor is as important as the hardware part, because through this interface, the final user interacts with the induction motor to perform the faults diagnosis. For instance, in Figure 13a, we observe the main screen template used to navigate between four action menus: the image acquisition tab, the ROI selection tab, the PCA analysis tab, and the automatic diagnosis Table. In first place, the image acquisition tab allows the user to execute an action to capture the thermal image, such as recording a video, taking a picture, saving the image, assigning a name to the saved file, and defining the color pallet for visualizing the temperature profile. In second place, the ROI selection tab allows the user to specify some important parameters for both system acquisition and data processing (Figure 13b). For instance, the sampling frequency and the acquisition time for capturing the thermal image, as well as the power supply frequency of the induction motor, are defined here. Additionally, the user can select between ROI1, ROI2, and ROI3 to perform the thermographic analysis, and if the analysis is for rolling bearings or the gearbox through the sliding button. However, the possibility of performing thermographic analysis for combined faults in bearings and gearbox is enabled by ticking the option of combined analysis from the blue round checkbox. Finally, the last two tabs, PCA analysis and automatic diagnosis, allow the user to observe the processing of the thermal image, showing the classes (clusters) found associated with the fault conditions in the case of the PCA analysis, or the final diagnosis semaphore indicated by the FFBNN in the case of the automatic diagnosis.

## 4. Infrared Thermography-Based Smart Sensor Tests and Results

**Figure 14:**
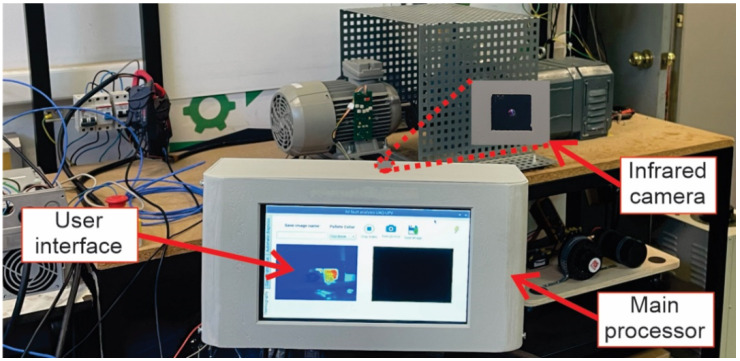
Real test bench used to validate the smart sensor based on infrared thermography.

In this section, the results obtained for different case studies will be presented, described, and discussed. The case studies correspond to the thermographic analysis performed by the smart sensor through the PCA analysis tab for the three ROIs on the coupling part, the body of the motor, and the back part of the motor. Additionally, case studies for the fault classification through the automatic diagnosis tab are presented, considering single and combined fault classification. For all the case studies, the thermal images were captured every 10 s (frame rate) for 30 min during the steady state of the motor operation, generating a total of 180 thermal images, of which 126 (70%) were used for training and 54 (30%) for testing. Thus, Figure 14 shows how the smart sensor based on infrared thermography is applied to detect and diagnose faults on the bearings and the gearbox of the induction motor described in the “Experimental test bench section”. 

### 4.1. Results of the PCA Analysis in the ROIs

**Figure 15:**
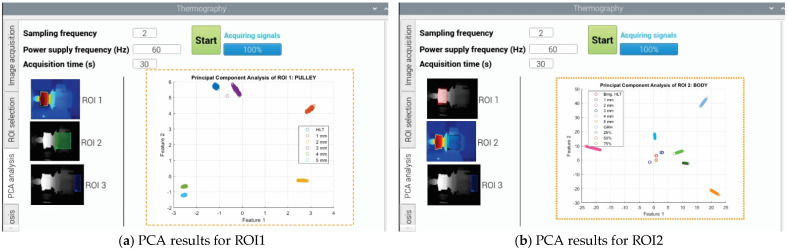

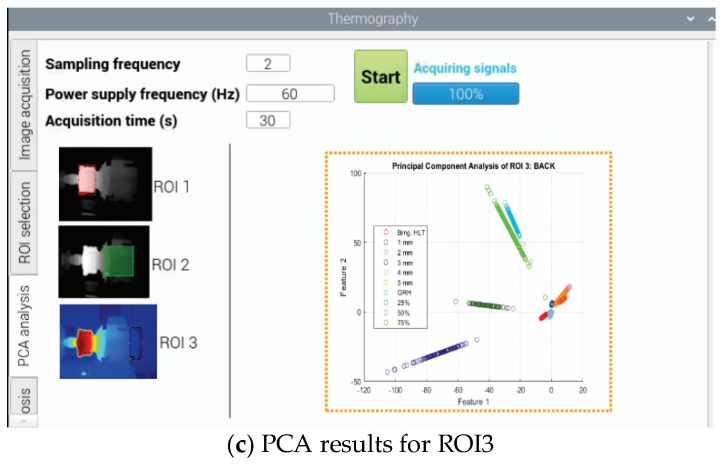
Results observed in the tab PCA analysis for the selected ROI for outer-race faults in the rolling bearings and gear wear into the gearbox of the induction motor, in (**a**) for the ROI1, in (**b**) for the ROI2, and in (**c**) for the ROI3.

Now, regarding the PCA analysis, three case studies will be addressed: PCA on the ROI1, ROI2, and ROI3. Therefore, Figure 15a depicts a plot of the two-dimensional representation by clusters, in which we observe five fault conditions and the healthy condition, clearly separated, for the rolling bearing element in the ROI1. That means that in the coupling part of the induction motor, not only can the gearbox be placed, but also a coupling based in rolling bearings. In this case, the motor is coupled with the load through a pulley, and the element of interest was the rolling bearing, which was replaced in order to have the healthy condition and the outer-race fault with gradualities (holes of 1 mm, 2 mm, 3 mm, 4 mm, and 5 mm). Furthermore, the processing time to achieve the results is 32 s, with an accuracy of 100% without overfitting, since the clusters are widely separated. In the case presented by Figure 15b, the plot shows the clusters of the faults related to the rolling bearing and the gearbox in the ROI2. Here, the ROI2 corresponds to the body of the motor, and it is demonstrated that the problems in the rolling bearings and the gearbox of the coupling zone near to the motor can be correctly detected. The clusters in this plot indicate that the healthy condition of the bearing, its corresponding five fault conditions in the outer-race, the healthy condition of the gearbox, and its three fault conditions of gear wear (backlash at: 25%, 50%, and 75%) are clearly grouped. The processing time in this case is 19 s, with an accuracy of 100%. Finally, the plot of Figure 15c presents the last case of PCA, showing the clusters of the faults related to the rolling bearing and the gearbox in the ROI3. In this case, the ROI3 corresponds to the back zone of the induction motor. This last plot shows the clusters for the same 10 conditions, as in the ROI2, but here, the grouped data are slightly overlapped for some situations. The processing time achieved in this case is 5 min, with an accuracy of 96.1%. 

From Figure 15, some observations can be made; for instance, the accuracy of the results will depend on the ROI selected. It must be chosen carefully, but the advantage of the smart sensor functionality is also clear, since the other two ROIs are capable of accurately diagnosing fault conditions. Even for the ROI3 in the zone with the lower performance, the obtained results are good. In general, the processing time for diagnosis is low; in the lower and higher values, 19 s and 5 min are required, respectively. Nevertheless, the average processing time is approximately 140 s; it will depend on the quality of the thermal images acquisition, the ROI selected, and the fault conditions.

### 4.2. Results of the FFBNN Fault Diagnosis

**Figure 16:**
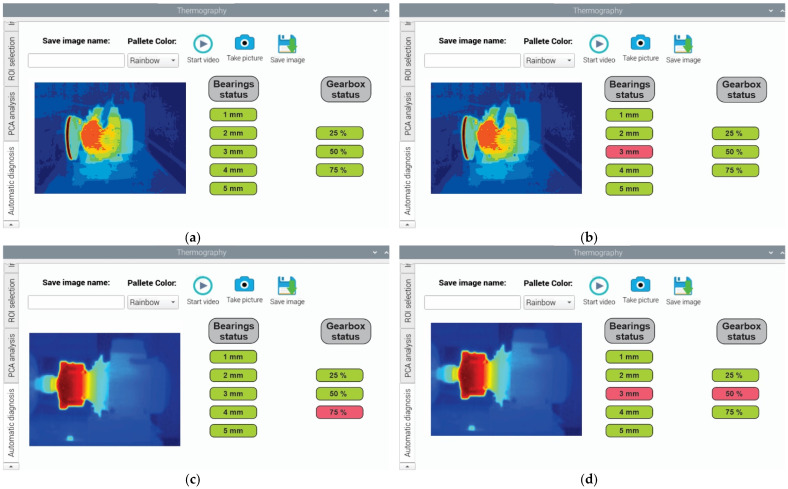
Results observed in the tab “Automatic diagnosis” for the rolling bearings and the gearbox of the induction motor, in (**a**) healthy conditions, in (**b**) outer-race fault with severity of 3 mm, in (**c**) gear wear with severity backlash of 75%, and in (**d**) combined fault as outer-race fault and gear wear with severities of 3 mm and backlash of 50%, respectively.

In relation to the automatic diagnosis through the FFBNN, four case studies are addressed: healthy conditions (no faults present), outer-race fault of the rolling bearing, gear wear inside the gearbox, and combined faults (outer-race and wear in the gear). Thus, Figure 16a displays the diagnostic report when the analysis finds that no faults are present in the rolling bearing or in the gearbox of the induction motor in form of a semaphore. As can be noticed from this figure, when there is no occurrence of faults, every condition considered appears colored in green. Another case is observed in Figure 16b; here, an outer-race fault is found in the bearing element, with severity of 3 mm (graduality in the condition). From this figure, it is noticed that when a fault occurs, the semaphore of the respective condition will be colored in red. Similarly, another case is observed in Figure 16c; on this occasion, wear in the gear is found inside the gearbox, with severity of 75%. Additionally, this condition’s severity is colored in red, while the remaining conditions appear in green. Finally, the last case study is observed in Figure 16d with a multiple fault condition, since the outer-race fault and wear in the gear are detected at the same time. In this last figure, it is noticed that the severities of the fault conditions are 3 mm in the rolling bearing and 50% in the gearbox, which are marked in red, and the remaining conditions are marked in green. The computing time spent for the final diagnosis in the semaphore may vary depending on the type of fault diagnosed, single or multiple, but on average, 140 s are required for performing the diagnosis.

Figure 16 allows us to remark that the smart sensor has flexibility and can be adjusted to the problem requirements, since single and multiple or combined faults can be detected and classified. Of course, the accuracy and precision of the semaphore of fault conditions (diagnostic report) will depend on the previous stages performed by the smart sensor; for instance, the quality of the primary infrared sensor, the data preprocessing and processing, the PCA analysis, and the FFBNN structure. Finally, although the smart sensor allows results to be obtained online, these results can also be obtained through the interface and an output port, allowing us to observe the diagnosis offline using a PC.

### 4.3. Summary of the System Characteristics and Prospective

In summary, the smart sensor overcomes the drawbacks faced by classical methodologies based on infrared thermography, and several advantages can be highlighted as follows: it is a proprietary system that performs fault diagnosis based on an infrared primary sensor and in processing boards for applications in field; the data do not need to be extracted and evaluated offline, because they are processed in the smart sensor. It integrates and an infrared camera and microcontroller units of low cost compared with the commercial monitoring systems; because it is a proprietary system, the functionality can be scaled by modifying the implemented algorithms; the infrared camera FLIR LEPTON 3.5 used has calibrated radiometric output and automatic temperature compensation. It transfers data to other external device, in addition to the graphical visualization in the smart sensor. However, some disadvantages of the smart sensor are listed as follows: the system performs the fault diagnosis of rolling bearings and the gearbox, but it can be expanded to detect more faults in other elements into a kinematic chain that reflect a change in the temperature as a consequence; only the thermography analysis is implemented, but other physical variables could be added through sensors for data fusion to complement the analysis. At the moment, the three ROIs used are predefined, but that could be improved through the automatic generation of ROIs.

## 5. Conclusions

This paper presents the structure and development of a smart sensor based on infrared thermography for faults diagnosis of associated elements of the induction motor, such as the rolling bearings and the gearbox. The structure design is modular, with simplicity and open architecture, which means that its functionality can be easily expanded and improved. Additionally, the hardware–software design enables future improvements and updates to the system, since all the electronic technologies are prone to obsolescence. Now, regarding the modular structure, the importance of the infrared sensor relies on characteristics such as its low cost and its small dimensions (micro module), even considering that it is a commercial camera. It worth mentioning that the infrared micro camera is a primary sensor, which means that it only provides raw data that will need interpretation, and this makes it adequate for integration into the smart sensor. Other reported works use high-cost commercial cameras, making them limited solutions in many cases. The accuracy of the smart sensor fault diagnosis will depend on the correct functioning of every module described; for instance, preprocessing is necessary to improve the quality of the information provided by the primary sensor, since the image resolution is not reduced but incremented, facilitating the definition of the ROIs. Along the same lines, adequate ROIs will provide zones where the faults are visible as a temperature increase, but they do not indicate the type of problem, or its graduality. In order to correctly detect the fault in the system, adequate features need to be computed, although there are several domains for analyzing data; the statistical features are quite well understood ones with easiness of implementation and low computational burden requirements. Additionally, among the different statistical features, several could reflect the type of fault in the data of a thermal image; for that reason, many indicators must be obtained. In counterpart, if several of the indicators provide redundant information, then meaningful features can be discriminated through the PCA, yielding only those features strongly related to the fault conditions. In this way, the PCA can be considered a preclassification stage, facilitating the final classification of the fault through a FFBNN that will provide accurate faults categorization. Finally, the diagnosis and PCA analysis can be visualized in the user interface of the smart sensor, making the system dynamic and intuitive, with the additional characteristic that the diagnostic report can be obtained through an output port, facilitating its visualization offline via the PC. Last, but not least, some limitations mentioned in the last subsection, as disadvantages of the system, will be considered as areas of opportunity that could be addressed to improve the system’s characteristics. Therefore, prospectively, the smart sensor will be expanded in functionality in software and hardware; for instance, considering more types of faults associated with changes in temperature, such as short-circuits in windings, power quality issues, etc. In this sense, other primary sensors can be added to the smart sensor, such as current, vibration, and stray flux, among others, increasing the variety of faults that could be analyzed, such as unbalance, misalignment, etc. Additionally, the possibility of using infrared cameras compatible with smartphones will be analyzed according to whether the application has technical and economic viability or not. Additionally, we will consider the requirements of keeping the cost low, having enough computational resources, easiness of software development, and open architecture.

## Data Availability

Not applicable.
